# Changing or stable? The effects of adolescents' social media use on psychosocial functioning

**DOI:** 10.1111/cdev.14207

**Published:** 2024-12-11

**Authors:** J. Loes Pouwels, Ine Beyens, Loes Keijsers, Patti M. Valkenburg

**Affiliations:** ^1^ Behavioural Science Institute Radboud University Nijmegen The Netherlands; ^2^ Amsterdam School of Communication Research University of Amsterdam Amsterdam The Netherlands; ^3^ Department of Psychology, Education and Child Studies Erasmus University Rotterdam The Netherlands

## Abstract

To better understand the effects of social media use on adolescents' psychosocial functioning, this study examined the temporal stability of social media effects across two separate 3‐week experience sampling methodology (ESM) studies conducted 6 months apart in 2019 and 2020. Participants were 297 adolescents (*M*_age_ = 14.1 years, SD = 0.7, 58.9% girls; 41.1% boys; 0.1% other; 97% Dutch) who completed 126 momentary questionnaires on social media use, affective well‐being, self‐esteem, and friendship closeness in each of the two ESM studies. The effects of social media on psychosocial functioning had low rank‐order stability (*r* = .05–.25). Findings indicated that the effects of social media use varied not only between individuals but also changed substantially within individuals over time.

**Abbreviations**
ESM: Experience Sampling Methodology
DSMM: Differential susceptibility to media‐effects model
OSF: Open Science Framework

## INTRODUCTION

Developmental psychology has a revived interest in understanding the heterogeneity of individual responses to environmental influences (Bolger et al., 2019; Keijsers & van Roekel, 2019; Lerner & Lerner, 2019). A noteworthy shift is occurring from traditional nomothetic studies, which generalize trends across populations, to idiographic or person‐specific approaches that unravel how psychosocial phenomena vary from person to person. The shift toward idiographic methods extends into examining social media effects on psychosocial functioning, defined as the within‐person changes in psychosocial functioning resulting from adolescents' use of social media (Valkenburg et al., 2016). Psychosocial functioning encompasses various developmental outcomes, such as forming and maintaining close friendships (Furman & Buhrmester, 1985), and acquiring self‐esteem (Orth & Robins, 2014), which are important developmental tasks in adolescence (Sullivan, 1953). Like affective well‐being, self‐esteem, and friendship closeness are key components of psychosocial functioning (Kernis, 1993; Ryff, 1989). Therefore, we specifically focused on affective well‐being, self‐esteem, and friendship closeness as indicators of psychosocial functioning.

Over the last decades, hundreds of studies have been conducted on social media effects on psychosocial functioning, of which the vast majority are cross‐sectional in nature (Marciano et al., 2024; Valkenburg et al., 2022). More recently, researchers have started to study longitudinal relations and to separate between‐person from within‐person variances in random‐intercept cross‐lagged panel designs (Coyne et al., 2020; Orben et al., 2019). Although insightful, these studies yield average within‐person effects at the sample level, which can only be generalized to any individual within the sample at most timeframes if assumptions of ergodicity hold, namely, that groups are composed of homogeneous individuals and that underlying processes are stable over time (i.e., stationary) (Adolf & Fried, 2019; Molenaar & Campbell, 2009).

Theories on idiographic social media effects propose that the homogeneity assumption does not hold because different adolescents experience different effects of social media on their psychosocial functioning depending on relatively stable and transient dispositional, developmental, and social contextual factors (Beyens et al., 2020; Valkenburg & Peter, 2013). Recently, several experience sampling methodology (ESM) studies have revealed substantial variations among adolescents in how their social media use relates to their psychosocial functioning (e.g., Beyens et al., 2021; Griffioen et al., 2022; Pouwels et al., 2021a; Rodriguez et al., 2021; Valkenburg, Pouwels, et al., 2021). The impact of social media use on adolescents' psychosocial functioning can be beneficial for some, harmful for others, and negligible for another group of adolescents (Verbeij et al., 2022). Thus, considerable variation exists among adolescents in the type, magnitude, and signs of the social media effects they experience.

Although earlier work suggests that the effects of social media use are heterogeneous rather than homogeneous (Valkenburg, Beyens, et al., 2021), the stability of these effects is an unanswered question. Previous research on person‐specific social media effects predominantly involved single ESM studies lasting one to 3 weeks (e.g., Beyens et al., 2021; Griffioen et al., 2022; Marciano, Driver, et al., 2022; Pouwels et al., 2021a; Rodriguez et al., 2021; Valkenburg, Pouwels, et al., 2021). Consequently, further evidence is needed to determine whether person‐specific social media effects are dynamic or stable.

Examining the stability of social media effects is important from a theoretical point of view. Dynamic system theories suggest that adolescents' psychosocial functioning is dynamic (Granic, 2005; Smith & Thelen, 2003) and undergoes periods of transition and change. Likewise, social media effects on psychosocial functioning could emerge, change, and disappear over time. Clarifying the stability of social media effects may aid social media effects researchers in understanding among whom, when, and why social media use influences psychosocial functioning. Further, it also has high practical relevance. In psychology, for instance, ESM data are used to derive person‐specific networks of symptoms, and interventions can be targeted to this unique pattern (Bringmann, 2021). Recent person‐specific studies on social media effects classified adolescents into “positive susceptibles,” “negative susceptibles,” and “non‐susceptibles” (Valkenburg, Beyens, et al., 2021). It has been argued that this distinction could be used to adequately target prevention and intervention strategies among negatively susceptible adolescents. However, this assumes that the effects are stable and that prevention and intervention would only work if the classification of adolescents into these susceptibility subgroups would hold over time.

Therefore, the aim of the present study is to investigate the level of stability and change in the effects of social media on an adolescent's psychosocial functioning (i.e., affective well‐being, self‐esteem, friendship closeness) across two separate 3‐week ESM studies conducted 6 months apart. We specifically focus on Instagram, Snapchat, and WhatsApp as these were the most popular social media among Dutch adolescents at the moment of data collection (van Driel et al., 2019).

### Theoretical explanations for the stability of social media effects

Enhancing our comprehension of the stability of social media effects holds the potential to refine media effect theories. Theoretically, a crucial next step involves unraveling the reasons behind the variability in social media effects among adolescents (Valkenburg et al., 2022). Grasping the driving forces behind social media effects is crucial to construct theoretical explanations for the heterogeneity in social media effects across adolescents. The (DSMM) (Valkenburg & Peter, 2013) proposes that certain adolescents exhibit greater susceptibility to social media effects than others due to a distinctive combination of relatively stable and transient dispositional, developmental, and social contextual factors. Stable factors encompass characteristics of the adolescents or their environment that tend to stay the same over extended periods of time, such as gender, social comparison orientation, developmental phase, or parental attachment (Buunk & Gibbons, 2006; Chris Fraley, 2002; Valkenburg & Peter, 2013). If the results of the current study show that media effects persist when adolescents participate in a second ESM Study 6 months later, we may conclude that stable characteristics likely play a pivotal role.

In contrast to stable characteristics, transient factors can fluctuate over shorter durations, ranging from days to weeks or months, rendering change in social media effects. These factors may include mood swings, leisure activities, relationship dynamics, or external influences like the COVID‐19 pandemic (Boele et al., 2022; Granic, 2005; Kuper et al., 2023; Maciejewski et al., 2019; Smith & Thelen, 2003). To some extent, these factors may affect whether adolescents are positively or negatively susceptible to social media use. For instance, positive social media effects may be experienced during romantic involvement, while negative effects may emerge post‐breakup (Fox et al., 2021). In addition to transient dispositional, developmental, and social contextual factors discussed in the DSMM, adolescents' social media use content may also fluctuate over time. For example, exposure to supportive social media posts and upward social comparison content could positively impact adolescents, whereas negative posts, risky self‐presentation, downward social comparison content, and online social exclusion might have adverse effects (Nesi et al., 2018; Orben et al., 2024). As transient factors fluctuate over time, we propose that adolescents' psychosocial functioning may be positively affected by social media use in specific periods but negatively in others (Valkenburg & Peter, 2013). If our results show that the recurrence of social media effects is not observed when adolescents participate in a second ESM Study 6 months later (Bolger et al., 2019), we can likely conclude that transient factors that may change over multiple months predominantly drive social media effects.

### A dynamic perspective on the stability of social media effects

Many adolescents experience problems with their psychosocial functioning at some point during adolescence. However, for most of them, these problems are transient, and a drop in psychosocial functioning may even serve as a motivator to address and resolve these issues (Granic & Patterson, 2006; Slater, 2007; Smith & Thelen, 2003). Dynamic system theories of development and Slater's reinforcing and downward spiral models posit that in the long term, stability in psychosocial functioning is maintained due to the interplay of positive (i.e., reinforcing spirals) and negative feedback loops (Cacioppo & Cacioppo, 2018; Granic, 2005; Slater, 2007).

In the context of social media use and psychosocial functioning, social media effects can amplify over time, reflecting a positive feedback loop, or stabilize over time, reflecting a negative feedback loop. Positive feedback loops may occur when increases in psychosocial functioning after using social media encourage adolescents to use it even more, further enhancing their psychosocial functioning (i.e., upward spirals). Interestingly, positive feedback loops can also arise from negative effects: excessive social media use can lead to social isolation, prompting further online engagement in a bid to connect, which only exacerbates the isolation (i.e., downward spirals). Conversely, negative feedback loops occur when the effects stabilize; for instance, excessive social media use may eventually reduce real‐life interactions and lower psychosocial functioning., which may motivate adolescents to reinvest in face‐to‐face interactions, decreasing their social media use and mitigating negative impacts. Thus, these intertwined feedback loops illustrate the complex dynamics of social media's impact on adolescent psychosocial functioning.

This interplay of positive and negative feedback loops may stabilize mean levels of psychosocial functioning in the long term. Only by following adolescents over extended periods of time, it is possible to investigate the potential impact of feedback loops and to find out whether (positive or negative) effects of social media use experienced in a certain period are predictive of the same effects experienced in later periods. Therefore, to fully understand how adolescents' social media use affects their psychosocial functioning, it is essential to gain insight into the longer‐term stability of their social media effects over multiple months. In the present study, we therefore examined the stability of social media effects across two ESM studies with a 6‐month time interval in between.

### The stability and change of social media effects in times of COVID‐19

In the current study, participants were exposed to the COVID‐19 pandemic between two ESM studies. The outbreak of the COVID‐19 pandemic and the subsequent lockdown served as a natural experiment to investigate the potential impact of transient environmental factors on the longer‐term stability of social media effects. It is especially important to study this potential impact among adolescents, given that the COVID‐19 pandemic induced profound changes in adolescent psychosocial functioning. For example, adolescents' levels of depressive symptoms increased from before to after the COVID‐19 pandemic, while their life satisfaction decreased (Mansfield et al., 2022; von Soest et al., 2022).

Social media use may have partly compensated for the lack of in‐person interactions. During the COVID lockdown, Dutch adolescents' face‐to‐face contact was reduced from 8 to 2 hours per weekday on average (Keijsers & Bülow, 2021), while their average time spent on social media increased from 3 to 5.5 hours per day (Keijsers & Bülow, 2021). Using social media may have stimulated friendship closeness during the COVID‐19 pandemic (Hamilton et al., 2022; Marciano, Ostroumova, et al., 2022). However, some adolescents experienced adverse effects of their social media use on their psychosocial functioning during the COVID‐19 pandemic (Marciano, Viswanath, et al., 2022), for example, due to enhanced social comparison, fear of missing out, and COVID‐19‐induced stress (Hamilton et al., 2022; Marciano, Ostroumova, et al., 2022). Previous ESM studies examined adolescents' effects on psychosocial functioning before or during COVID‐19. However, it remains uncertain to what extent adolescents' individual social media effects changed across 6 months with a COVID‐19 lockdown in between. The primary aim of this study is to investigate the extent to which such changes occurred. If our results show that social media effects changed throughout the pandemic, we can likely conclude that social media effects are context‐dependent and subject to change over more extended periods of time, which would be an important consideration for social media effects theories and interventions.

### Assessing stability and change in social media effects

To understand the stability and change in social media effects, we adopt three indices commonly applied in personality psychology: rank‐order stability, mean‐level change, and individual‐level change (e.g., Roberts et al., 2001). First, the rank‐order stability reflects the test–retest correlation of adolescents' effects of social media use on psychosocial functioning between ESM studies 1 and 2. A high rank‐order stability coefficient indicates that individuals with the most pronounced positive (or negative) social media effects during ESM 1 continue to exhibit the strongest positive (or negative) effects during ESM 2. The rank‐order stability offers insight into the extent to which the relative ordering of individuals based on the effects of social media is sustained over time (Roberts et al., 2001). Second, the mean‐level change captures the overall difference in the means of social media effects observed during ESM studies 1 and 2 (Roberts et al., 2001). Together, the rank‐order stability and mean‐level change reveal the extent to which social media effects are stable, signifying whether they remain similar in both sign and magnitude (Roberts et al., 2001).

Whereas rank‐order stability and mean‐level change offer insight into the average stability and change of social media effects at the sample level, they do not shed light on patterns of change at the individual level. The third type of change, individual‐level change, delves into patterns of change experienced by individuals. When social media effects remain stable regarding their mean level but shift in rank order, some adolescents may become increasingly positively affected over time, while others may be more negatively affected (Nesselroade, 1991; Roberts et al., 2001). As these changes at the individual level may cancel each other out at the mean level, examining individual‐level change in addition to mean‐level change is crucial. By comparing person‐specific media effects in ESM studies 1 and 2, this type of change allows us to ascertain whether each adolescent's social media effects on psychosocial functioning have increased, decreased, or remained constant over time.

### The present study

To understand whether social media effects are changing or stable, the present study examined the rank‐order stability, mean‐level change, and individual‐level change of social media effects over time, specifically focusing on three indicators of psychosocial functioning: affective well‐being, self‐esteem, and friendship closeness. This study used data from a larger project on adolescents' social media use and psychosocial functioning, employing a measurement burst design. The overarching project compromised two three‐week ESM studies (i.e., ESM bursts) conducted half a year apart, during which adolescents' social media use, affective well‐being, self‐esteem, and friendship closeness were measured six times per day over 3 weeks. Notably, the COVID‐19 pandemic started between these two studies, providing a unique opportunity to examine potential shifts in social media effects on psychosocial functioning.

We developed research questions rather than hypotheses to investigate the stability of the effects of social media use on psychosocial functioning because this is the first study that combines and compares two ESM studies. Therefore, little is known regarding how stable media effects are over time, and our work is relatively exploratory. We investigated the following research questions: What are the rank‐order stability (RP1a) and mean‐level change (RQ1b) in the effects of social media on adolescents' psychosocial functioning across a time interval of 6 months, with a COVID‐19 lockdown in between? As media effects may change to a different degree across different adolescents, we also examined adolescents' individual‐level change with the following research question: For how many adolescents do the effects of social media use on their psychosocial functioning increase, decrease, or stay stable across a time interval of 6 months (RQ2)?

## METHOD

**FIGURE 1:**
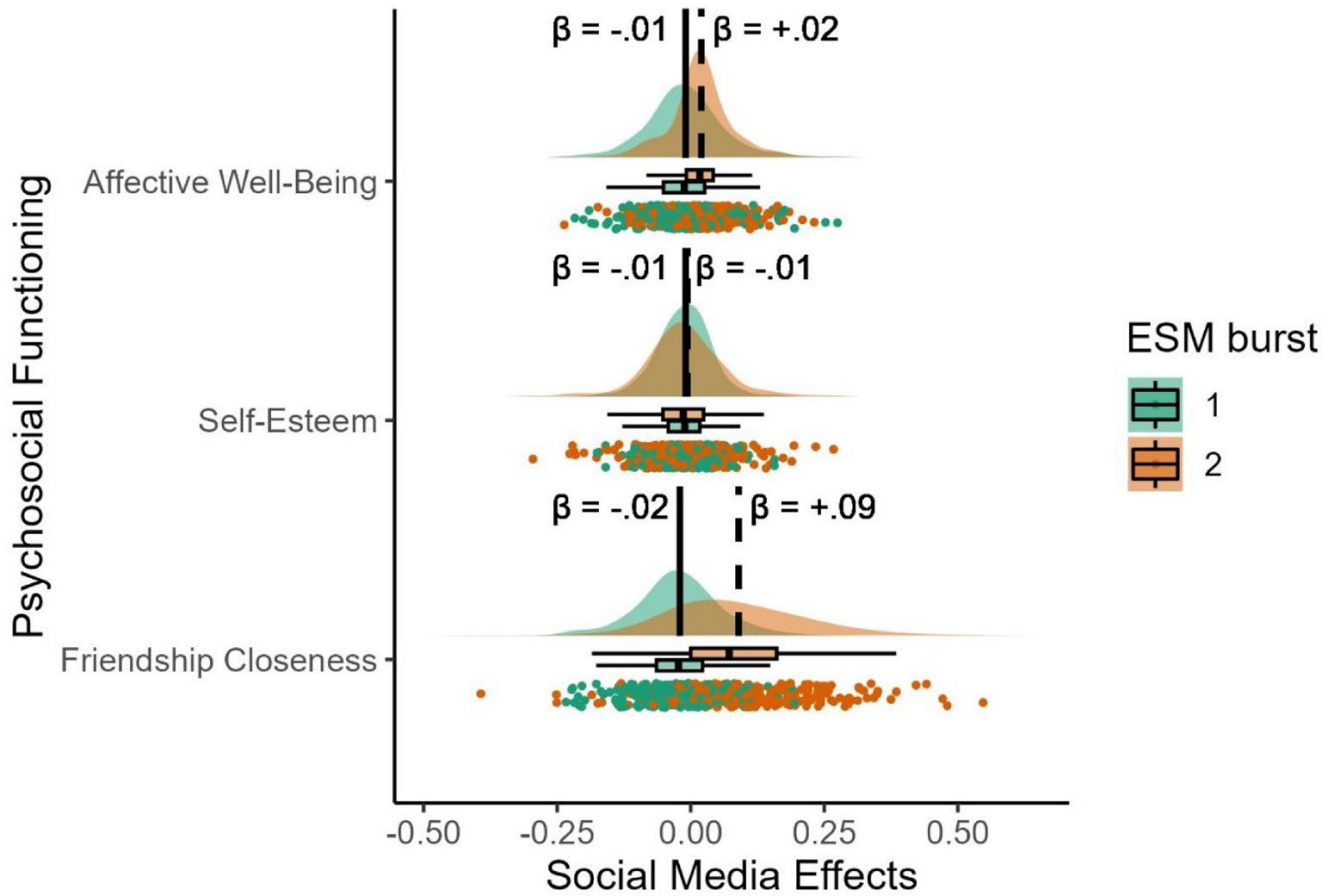
Raincloud plots of social media effects on three types of psychosocial functioning across two experience sampling methodology (ESM) studies (i.e., bursts). Standardized effect sizes are displayed on the *x*‐axis and the type of psychosocial functioning on the *y*‐axis. The density plots (i.e., “clouds”) represent a visualization of the distribution of the effect sizes, the vertical lines represent the mean scores (solid lines represent means of study 1; dashed lines the means of study 2), the “rain” represents the raw individual effect sizes, and the boxplots represent the median and 25% percentiles.

This study was part of a larger project on adolescents' social media use and psychosocial functioning executed from November 2019 (ESM Study 1) to June 2020 (ESM Study 2). The study had a measurement burst design, consisting of two ESM bursts 6 months apart, 16 biweekly surveys, tracking of social media use during ESM Study 2, and data donations (see OSF Figure 1; https://osf.io/wnfh8). The first ESM study occurred in 2019 before the COVID‐19 pandemic emerged. From March through May 2020, there was a COVID‐19 lockdown. Adolescents had to stay at home as much as possible, secondary schools were closed, and social events were restricted. The second ESM study was conducted in June 2020, right after the first COVID‐19 lockdown ended and adolescents could return to school.

The effects of social media use on adolescents' well‐being (Beyens et al., 2021; Verbeij et al., 2022), self‐esteem (Valkenburg, Beyens, et al., 2021; Valkenburg, Pouwels, et al., 2021; Verbeij et al., 2022), and friendship closeness (Pouwels et al., 2021a, 2021b; Verbeij et al., 2022) have already been examined as part of our previous studies, based on either the first or second ESM study. In the present study, we will extend our previous research by investigating three types of change and stability in social media effects across the two ESM studies (i.e., rank‐order stability, mean‐level change, and individual‐level change).

### Participants and recruitment

The final sample of this study consisted of 297 13‐ to 16‐year‐old adolescents (58.99% girls; 41.1% boys; 0.1% other) with a mean age of 14.08 (*SD* = 0.70 years) at the start of the project. Of all participants, 37% were enrolled in lower prevocational secondary education (VMBO), 34% in intermediate general secondary education (HAVO), and 30% in academic preparatory education (VWO). The sample accurately represented the municipality of the participating school in the south of the Netherlands regarding country of birth because 97% of the adolescents were born in the Netherlands and self‐identified as Dutch (Statistics Netherlands, 2020).

### Procedure

We invited all 745 students in Grades 8 and 9 from a large secondary school. In total, 400 students received informed consent from their parents, and 388 provided informed assent. As some students dropped out during the study, the sample of the first ESM study consisted of 387 participants, and the second study sample was 312 participants. We excluded three out of 312 participants from the analyses as they did not meet the eligibility requirement to use Instagram, WhatsApp, or Snapchat more than once per week, according to the pre‐ESM survey. In addition, 12 participants were excluded because they did not report any social media use during ESM 2. Therefore, the final sample of this study consisted of 297 Instagram, WhatsApp, or Snapchat users who completed both ESM studies.

During each ESM study, which lasted 21 days, adolescents completed six surveys per day on their social media use, well‐being, self‐esteem, and friendship closeness (126 measurements in total) at random time points within a fixed time interval. A detailed overview of the questionnaires, notification schemes, response windows, and reminders is provided on the Open Science Framework (OSF; https://osf.io/tbdjq). For each questionnaire that was completed, we compensated adolescents with €0.30. In addition, we organized a raffle each day in which four participants who completed all six questionnaires the previous day could win €25. On average, adolescents completed 77% of the surveys in ESM study 1 (*M* = 97 surveys, *SD* = 19.56) and 58% in study 2 (*M* = 73.33 surveys, SD = 19.56). According to our power analyses, we therefore had enough power to detect small social media effects (see https://osf.io/ar4vm/).

### Measures

#### Social media use

During each ESM study, we measured adolescents' time spent using the three most popular social media platforms among Dutch adolescents in the year of data collection: Instagram, Snapchat, and WhatsApp (van Driel et al., 2019). During ESM 1, we measured adolescents' time spent on social media in the hour before the ESM survey with the active private (3 items), passive private (3 items), and passive public (2 items) subscales of the ESM Social Media Use Questionnaire (E‐SMUQ; Beyens et al., 2021). These values were summed.

In the second ESM study, we asked adolescents to sum themselves how much time they had spent using Instagram, Snapchat, and WhatsApp in the past hour. In both ESM studies, adolescents responded to each item on a scale from 0 to 60 minutes with 1‐minute intervals. Following the procedure of Valkenburg, Beyens, et al. (2021), we created a sum score of adolescents' total time spent on social media per assessment. Sum scores that exceeded 60 minutes were recoded into 60. Sum scores were divided by 10 to make their range comparable to the range of the psychosocial functioning measures (i.e., 0–6; Valkenburg, Beyens, et al., 2021). The intra‐class correlations (ICCs) were .46 in ESM study 1 and .51 in ESM study 2. These ICCs indicate that 46% and 51% of the variance in momentary social media use during ESM studies 1 and 2 could be explained by stable between‐person differences, while the remaining variances of 54% and 49% were due to within‐person differences and error.

#### Affective well‐being

Like previous ESM research that confirmed the validity of a single‐item ESM measure of well‐being (Beyens et al., 2020), we measured affective well‐being with one item: “How happy do you feel right now?”. Adolescents were asked to answer the question on a 7‐point scale ranging from 0 (“not at all”) to 6 (“completely”), with 3 (a little) as the midpoint. The ICC was .44 during ESM study 1 and .56 during ESM study 2.

#### Self‐esteem

Self‐esteem was measured with one item from the Rosenberg self‐esteem scale (Rosenberg, 1965; Valkenburg, Pouwels, et al., 2021): “How satisfied about yourself do you feel right now?”. Adolescents responded on a 7‐point scale, ranging from 0 (“not at all”) to 6 (“completely”), with 3 (“a little”) as the midpoint. Previous research has supported the validity of single‐item measures of self‐esteem (Robins et al., 2001). The ICC was .46 during ESM study 1 and .53 during ESM study 2.

#### Friendship closeness

Friendship closeness was measured by asking adolescents, *“How close to your close friends do you feel right now?”* (Lee, 2009; Pouwels et al., 2021a). Adolescents responded on an answer scale ranging from 0, “not at all,” to 6, “completely,” with 3, “a little,” as the midpoint. The ICC was .41 during ESM study 1 and .50 during ESM study 2. Pouwels et al. showed that adolescents' understanding of the concept of friendship closeness aligned with the social provisions of Furman and Buhrmester (1985).

### Statistical analyses

Unless otherwise indicated, we followed our preregistered analysis plan (https://osf.io/zftmv/) to examine stability and mean‐level change (RQ1) and individual‐level change (RQ2).

#### Stability and mean‐level change

To determine the stability (RQ 1a) and mean‐level change (RQ 1b), we first estimated the effects of social media use on affective well‐being, self‐esteem, and friendship closeness with two series of autoregressive lag 1(AR1) Dynamic Structural Equation Models (DSEM) in Mplus 8.5 (Asparouhov et al., 2018). We preregistered that we would estimate the effects of social media use on all three indicators of psychosocial functioning in a single model (for model specifications, see https://osf.io/btvuw).

At the within‐person level, we included the autoregressive effects of well‐being, self‐esteem, and friendship closeness to control for psychosocial functioning at each previous assessment. In addition, we estimated the average within‐person effects of social media use on the three outcome measures. We followed McNeish and Hamaker (2020) by predicting each indicator of psychosocial functioning from social media use measured at the same measurement occasion because we measured social media use with regard to “the past hour” and psychosocial functioning “right now.” The different time spans of our measures, therefore, implied temporal precedence. We set the time interval to 2 hours using the T‐interval statement in Mplus to rescale time into two‐hour increments so that we could consistently interpret the autoregressive paths as the carryover from 2 hours prior to the assessment (McNeish & Hamaker, 2020). We person‐mean‐centered all within‐person variables so that within‐person estimates are automatically controlled for all between‐person variation in the estimation of the model. Using the standardized (cluster) function in Mplus, this model enabled us to determine each adolescent's person‐specific effect of social media use on well‐being, self‐esteem, and friendship closeness.

At the between‐person level, we specified between‐person variance around adolescents' average levels of well‐being, self‐esteem, and friendship closeness (i.e., random intercepts), the average within‐person effects of social media use on well‐being, self‐esteem, and friendship closeness (i.e., random slopes), and the autoregressive effects. Between‐person variables were grand‐mean‐centered.

As the preregistered models in which we combined the three indicators of psychosocial functioning did not converge, we simplified our analysis strategy by following our preregistration. Specifically, we estimated the effects of social media use on each indicator of psychosocial functioning in separate models. This resulted in six models (3 outcomes measures * 2 ESM studies).

The output of each model included the (a) overall model results and (b) person‐specific results for each adolescent, including a standardized social media effect that represents the within‐person change in psychosocial functioning that results from his or her social media use and a posterior standard deviation of the effect. The overall model results are presented at the Open Science Framework (https://osf.io/btvuw) because this article specifically focuses on the change and stability of person‐specific media effects, and the overall model results have already been published in our previous work. To get insight into the change and stability of social media effects, we imported the estimated standardized person‐specific effects from the Mplus DSEM output into R (R Core Team, 2020). Subsequently, we examined the rank‐order stability, mean‐level change, and individual‐level change.

#### Rank‐order stability

The rank‐order stability (RQ1a) was determined by computing a between‐person correlation between the person‐specific social media effects obtained from ESM 1 and ESM 2 using the Statistical Functions for Regression Models (sjstats) package (Lüdecke, 2021). We examined whether the rank‐order stability coefficients of the social media effects on the three different indicators of psychosocial functioning were significantly different from each other with comparisons of dependent nonoverlapping correlations using the cocor package (Diedenhofen & Musch, 2015).

#### Mean‐level change

The mean‐level change (RQ1b) in social media effects on affective well‐being, friendship closeness, and self‐esteem from ESM studies 1–2 was examined by comparing the mean effects with one paired sample *t*‐test per indicator of psychosocial functioning using the rstatix package (Kassambara, 2023). We reported Cohen's d as a measure of effect size and used α ≤ .05 to infer significance.

#### Individual‐level change

Individual‐level change (RQ2) in the effects of social media use on affective well‐being, friendship closeness, and self‐esteem from ESM studies 1–2 was examined by determining how many adolescents' social media effects had increased, decreased, or stayed the same. There is currently a lack of criteria to decide whether person‐specific effects are stable at the individual level. We therefore preregistered that we would determine whether adolescents experienced positive, negative, or non‐existent small media effects using the effect of .05 as the smallest effect size of interest. This effect size has been indicated as the smallest effect size of interest in the media effects literature (Meier & Reinecke, 2021), and effect size guidelines of autoregressive or cross‐lagged studies indicate that effects of .05 could be considered meaningful (Adachi & Willoughby, 2015; Orth et al., 2024). Subsequently, we examined for how many adolescents the effect remained consistently positive, negative, or non‐existent across both ESM studies.

## RESULTS

### Mean‐level change and stability in social media use and psychosocial functioning

Before we examined the stability and change in *social media effects* from ESM study 1 to ESM study 2, we inspected the stability and change in adolescents' *mean levels* of psychosocial functioning and social media use across the 3 weeks of each ESM study (see Table 1). Adolescents spent, on average, about 15 minutes on social media the hour before each ESM assessment. In addition, on average, adolescents experienced relatively high levels of affective well‐being, self‐esteem, and friendship closeness in each ESM study (*M* = 3.49–4.52; scale 0–6). There was no significant change in adolescents' mean level of psychosocial functioning and social media use from ESM study 1–2.

**TABLE 1:** Mean‐level change and rank‐order stability of mean levels of social media use and psychosocial functioning.

	ESM 1		ESM 2		Mean‐level change		Rank‐order stability^a^
	*M*	SD	*M*	SD	*t*_(296)_	Cohen's *d*	*r*
Social media use^b^	15.53	13.56	14.90	11.98	.85	0.05	.51*
Affective well‐being^c^	4.52	1.01	4.46	1.09	1.43	0.08	.73*
Self‐esteem^c^	4.14	1.11	4.16	1.06	−.40	−0.02	.68*
Friendship closeness^c^	3.49	1.30	3.52	1.29	−.43	−0.03	.72*

*Note*: The means of ESM 1 and ESM 2 did not differ significantly.

Abbreviation: ESM, experience sampling methodology.

^a^ We computed rank‐order stability with the Pearson correlation between ESM 1 and ESM 2.

^b^ Time spent on social media was measured on a scale of 0–60 min.

^c^ Each indicator of psychosocial functioning was measured on a scale ranging from 0 to 6.

* *p* < .001.

We found a high rank‐order stability of all three indicators of psychosocial functioning (see Table 1) because adolescents' levels of psychosocial functioning during ESM study 1 were strongly correlated with their levels of psychosocial functioning during ESM study 2 (*r* = .68 to *r* = .73; *p* values < .001). Adolescents' time spent on social media at ESM study 1 was also significantly and strongly correlated (*r* = .51, *p* < .001) to their time spent on social media at ESM study 2.

### Rank‐order stability, mean‐level change, and individual‐level change in social media effects on psychosocial functioning

As a next step, we examined the rank‐order stability (RQ1a) and mean‐level change (RQ1b) in the *effects* of social media on adolescents' psychosocial functioning across a time interval of 6 months. The raincloud plots in Figure 1 display the overlap in the distribution of social media effects between ESM studies 1 and 2 for each indicator of psychosocial functioning. The raincloud plots show the distribution and means (i.e., see vertical lines) of the effects of social media use on psychosocial functioning in the sample per ESM study. The difference in the mean effect of social media use observed during study 1 compared with study 2 was bigger for friendship closeness than for affective well‐being and self‐esteem, as indicated by a greater distance between the solid and dashed vertical lines. The distributions showed that during ESM study 2, the distribution of effects of social media use on well‐being was denser than during ESM 1, while for friendship closeness, the bell curve was much flatter. These findings suggest that while the effects of social media use on friendship closeness became more heterogeneous after the COVID‐19 lockdown than before the lockdown, the effects on affective well‐being became more homogeneous. The raincloud plots do not shed light on individual patterns of change. Means, standard deviations, *t*‐tests, and rank‐order stability coefficients are reported in Table 2.

**TABLE 2:** Mean‐level change and rank‐order stability of social media effects of social media use on psychosocial functioning.

	ESM 1		ESM 2		Mean‐level change		Rank‐order stability^a^
	*M*	SD	*M*	SD	*t*(296)	Cohen's *d*	*r*
Affective well‐being	−0.01	0.07	0.02	0.06	−5.52*	−0.32	.11
Self‐esteem	−0.01	0.05	−0.01	0.07	−0.14	−0.01	.25*
Friendship closeness	−0.02	0.08	0.09	0.13	−12.70*	−0.74	.05

Abbreviation: ESM, experience sampling methodology.

^a^ Rank‐order stability was computed with the Pearson correlation between social media effects at ESM 1 and ESM 2.

* *p* < .001.

**FIGURE 2:**
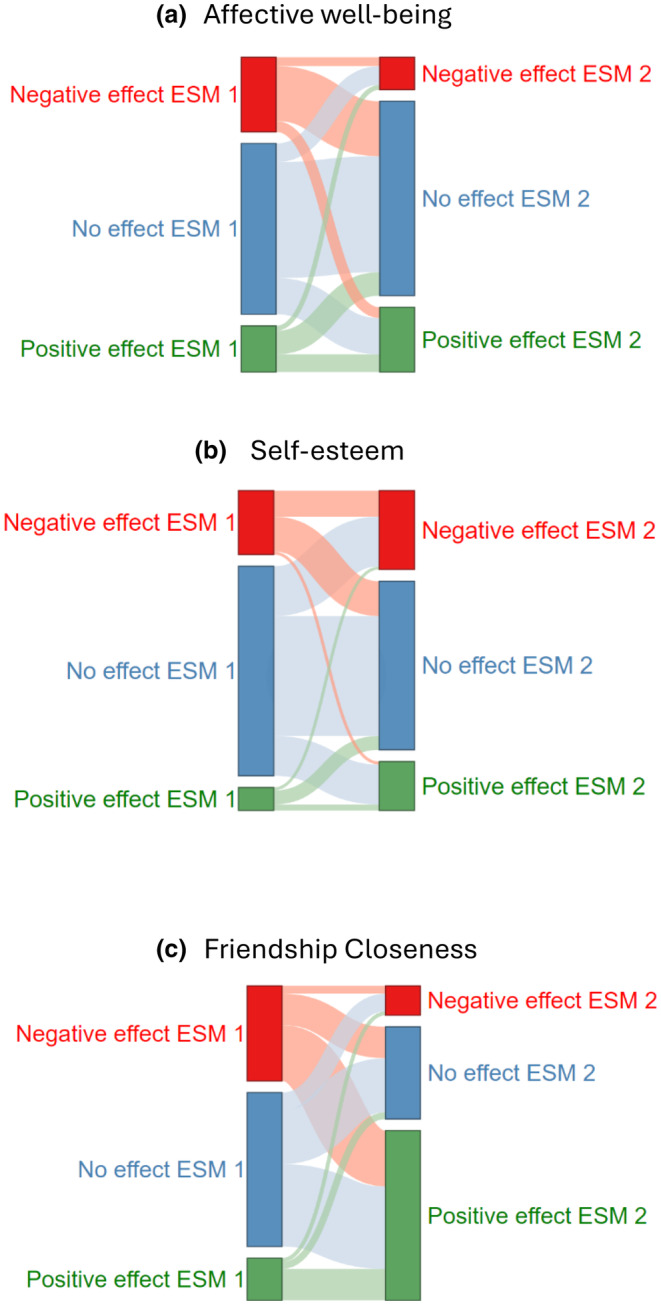
Sankey networks displaying individual‐level change in social media effects on psychosocial functioning from experience sampling methodology (ESM) 1 to ESM 2. The Sankey network displays subgroup classification flows between ESM 1 and 2 for participants with a negative effect (β ≤ −.05; red), a null effect (−.05 < β < .05; blue), and a positive effect (β ≥ .05; green) of social media on psychosocial functioning. An interactive HTML version of the Sankey Networks, including the absolute number of participants per node and link, can be found in OSF Supplement 1 (https://osf.io/3ej9h).

Further, we also examined for how many adolescents the within‐person effects of social media use on their psychosocial functioning became more positive, more negative, or were stable across 6 months (RQ2). For each ESM study, we classified adolescents into positive effect, negative effect, and no effect subgroups, using an effect size of β (−).05 as the cut‐off point based on earlier work (Meier & Reinecke, 2021). Subsequently, we examined among how many adolescents the classification remained similar or changed over time. Effects became more positive if adolescents were classified into a negative effect subgroup in ESM 1 and a no effect or positive effect subgroup in ESM 2 or if they were classified into a no effect subgroup in ESM 1 and a positive effect group in ESM 2. Likewise, effects became more negative if adolescents were classified into a positive effect group in ESM 1 and a no effect or negative effect group in ESM 2 or if they were classified into a no effect subgroup in ESM 1 and a negative effect group in ESM 2. The Sankey Networks in Figure 2 show how the effects of study 1 evolve into the effects of study 2.

#### Affective well‐being

RQ1a asked whether individuals who experienced the strongest positive (or negative) social media effects during ESM study 1 continued to display the strongest positive (or negative) social media effects during ESM study 2. The rank‐order stability was low (*r* = .11, *p* = .055) as the effects on well‐being were only weakly and not significantly correlated. The rank order of individual media effects was not maintained over time. Although there was a significant mean‐level change in the within‐person social media effects on affective well‐being (RQ1b see Table 2), both mean effects were non‐existent to small in both studies (*M*_β_ = −.01, *M*_β_ = .02), and there was quite some overlap in the distribution in the media effects (see Figure 1). The overall change could, therefore, be considered negligible to small. Together, the combination of a low rank‐order stability and negligible to small mean‐level change suggests that the effect of social media use on well‐being changed to a different degree across adolescents from ESM study 1 to ESM study 2. Thus, whereas the person's means of well‐being remained stable over time, the social media effects on well‐being did change.

We also found some individual‐level changes in the social media effects on affective well‐being (RQ2; Figure 2). The largest subgroup (40%) consisted of adolescents who experienced stable, nonsignificant, to small social media effects across the two studies. And 3% experienced stable negative effects, and 6% had stable positive effects. Among a small majority of adolescents (i.e., 51%), the effects of social media changed. Specifically, among 35% of the participants, the social media effects on affective well‐being became more positive (or less negative) from ESM studies 1–2. In comparison, among 16%, the effects became more negative (or less positive).

#### Self‐esteem

For self‐esteem, the rank‐order stability (RQ1b) was significant, as indicated by a positive correlation between the social media effects in ESM study 1 and ESM study 2 (*r* = .25, *p* < .001). Those more strongly affected by social media use in ESM 1 were also somewhat more strongly affected in ESM2. We did not find significant mean‐level change (RQ1b) because we replicated the effects and found a non‐existent to small within‐person effect of social media use on self‐esteem at both ESM study 1 and 2 (*M*_β_ = −.01, see Table 2) and the distribution of media effects was relatively similar (see Figure 1). These findings indicate some maintenance of the sign, magnitude, and rank order of adolescents' social media effects on self‐esteem over time.

Like the individual‐level findings for well‐being (RQ2), the largest subgroup (40%) consisted of adolescents who experienced stable nonsignificant to small social media effects on self‐esteem across the two ESM studies. About 9% experienced stable negative effects, and 2% had stable positive effects. The effect of social media use on self‐esteem changed over time for 49% of adolescents. Specifically, the effects became more positive (or less negative) among 26% of the participants and more negative (or less positive) among 23%.

#### Friendship closeness

The rank‐order‐stability (RQ1a) of social media effects on friendship closeness was low, as indicated by a nonsignificant small correlation between social media effects at ESM study 1 and 2 (*r* = .05, *p* = .400). The rank‐order stability coefficient of the effect on friendship closeness (*r* = .05) was significantly lower than that of the effect on self‐esteem (*r* = .25), *Z* = 7.52, *p* = .057, whereas the rank‐order stability coefficient of the effect on affective well‐being did not differ significantly from that of friendship closeness and self‐esteem. Figure 2 and Table 1 show that the average within‐person social media effect on friendship closeness significantly changed from ESM 1 to 2 (RQ1b). Specifically, social media use was positively related to friendship closeness in ESM study 2 (*M*_β_ = .09) but not in ESM study 1 (*M*_β_ = −.02). Together, these findings suggest that although, on average, adolescents experienced a more positive effect of social media use on friendship closeness during study 2 than 1, the rank‐order was not maintained.

Figure 2 shows that the links connecting nodes that are different across ESM waves (e.g., negative in ESM 1 and positive or no effect in ESM 2) were wider in the friendship closeness figure than in the affective well‐being and self‐esteem figures. This suggests that the individual‐level change in social media effects on friendship closeness was larger than the change in social media effects on affective well‐being and self‐esteem. Specifically, among 68% of the participants, the classification of social media effects on friendship closeness changed. For most adolescents (58%), the effects of social media use on friendship closeness became more positive over time. In total, 47% of the adolescents experienced a negative or non‐existent to small impact of social media on friendship closeness before COVID‐19 (ESM 1), but a positive effect after COVID‐19 (ESM 2). For only 15% of adolescents, the effect of social media use on friendship closeness became more negative from ESM studies 1 to 2.

### Exploratory analyses

We conducted exploratory analyses to compare individual‐level change across the three psychosocial functioning components and to explain the change in effects. First, we explored whether adolescents who experienced an over time change in social media effects for one component of psychosocial functioning experienced a similar change in social media effects for the other components of psychosocial functioning. For each adolescent, we first computed one difference score per component of psychosocial functioning by subtracting the social media effect at ESM 1 from the social media effect at ESM 2. Next, to examine to what extent the individual‐level changes in social media effects on affective well‐being, self‐esteem, and friendship closeness were related, we computed correlations between the difference scores for the three components of psychosocial functioning. Adolescents' individual‐level changes in social media effects on all three psychosocial functioning components were significantly correlated (*r* = .25 to 0.38, *p <* .001). These findings suggest that those adolescents who were likely to experience relatively more positive (or negative) social media effects on affective well‐being in ESM study 2 than 1 were also more likely to experience more positive (or negative) social media effects on self‐esteem and friendship closeness in ESM study 2 than 1.

Second, we were interested in potential explanations for the change in social media's effects on psychosocial functioning. We conducted exploratory analyses to examine whether dispositional, transient, and methodological factors could explain the heterogeneity in rank‐order stability. For each indicator of psychosocial functioning, we conducted four regression analyses. To examine the rank‐order stability, social media effects at ESM 1 were included as a predictor of social media effects at ESM 2 in each analysis. In addition to the social media effects at ESM 1, we predicted the social media effects at ESM 2 from gender (Table A1), level of social media use and psychosocial functioning (Table A2), COVID‐19 experiences (Table A3), and the posterior standard deviation of the social media effect (Table A4). We also examined the interactions between the social media effects at ESM 1 and gender, levels of psychosocial functioning and social media use, COVID‐19 experiences, and the posterior standard deviation of the effect, respectively. Significant interactions suggest that the rank‐order stability coefficient depended on one of the other variables of interest. The findings of these explorative analyses are discussed below.

In Table A1, we explored the role of gender because previous research revealed gender differences in social media effects on psychosocial functioning (Pouwels et al., 2021a). As the main and interaction effects for gender were insignificant, the rank‐order stability and mean‐level change did not depend on gender.

In Table A2, we examined how the stability and change depended on adolescents' levels of social media use and psychosocial functioning during ESM 1 because previous research found that social media effects are more positive for adolescents with lower levels of psychosocial functioning (Pouwels et al., 2021b; Valkenburg, Beyens, et al., 2021). The mean‐level change in social media effects on all three indicators of psychosocial functioning did depend on adolescents' mean level of psychosocial functioning during ESM 1. Specifically, compared with their peers, adolescents with lower levels of affective well‐being (β = −.14, *p* = .025), self‐esteem (β = −.38, *p* < .001), or friendship closeness (β = −.34, *p* < .001) during ESM 1 experienced a more positive social media effect on psychosocial functioning during ESM 2 while controlling for their social media effect during ESM 1. Adolescents who spent relatively little time on social media during ESM 1 had, as compared with their peers, a more positive social media effect on affective well‐being (β = −.12, *p* = .037) and friendship closeness (β = −.22, *p* < .001) during ESM 2. Regarding friendship closeness, the social media effects were more stable among adolescents who spent more time using social media during ESM 1 than among those who spent less time using social media, as indicated by a significant interaction between the social media effects and levels of social media use during ESM 1 (β = .13, *p* = .020).

In Table A3, we examined the role of adolescents' perceptions of positive and negative life changes due to COVID‐19 and whether they perceived changes in social media use due to the pandemic, given that adolescents' COVID‐19 experiences impact their development (Mansfield et al., 2022; Marciano, Viswanath, et al., 2022; Nunes et al., 2023; von Soest et al., 2022). Regarding affective well‐being and self‐esteem, we found that while controlling for the social media effects during study 1, more negative effects of social media use were experienced during study 2 among participants who indicated that COVID‐19 changed their social media use (β = −.13, *p* = .030 and β = −12, *p* = .046, respectively). Regarding friendship closeness, we found that participants who indicated that COVID‐19 hurt their lives experienced a more positive effect of social media use on friendship closeness during ESM 2 (β = .15, *p* < .012). The effect of friendship closeness on social media use was particularly stable among adolescents who indicated that COVID‐19 positively impacted their lives, as indicated by a significant interaction between the social media effects at ESM 1 and positive COVID‐19 experiences (β = .14, *p* = .020). This suggests that adolescents with a relatively positive social media effect on friendship closeness during ESM 1 were still likely to have a positive effect on friendship closeness during ESM 2, but only if they perceived that COVID‐19 positively impacted their lives.

In Table A4, we explored the effect of the uncertainty of each adolescent's social media effect on the stability. As part of the output of the DSEM model, each adolescent gets its own person‐specific social media effect along with a posterior standard deviation, which summarizes the uncertainty of this effect. For certain adolescents, the effects are more certain than for others, for example, because they completed more ESM questionnaires. A higher uncertainty around the effect may negatively impact the stability. Adolescents whose social media effect at ESM 1 was more uncertain had, as compared with their peers, a relatively more positive social media effect on friendship closeness during ESM 2 while controlling for their social media effect during ESM 1 (β = .18, *p* = .002). They also had a less stable social media effect on friendship closeness but not on self‐esteem and affective well‐being, as indicated by a significant interaction between the social media effect on friendship closeness at ESM 1 and the posterior standard deviation of this effect (β = −.12, *p* = .045).

## DISCUSSION

In the past decades, numerous studies have investigated the effects of social media use on adolescents' well‐being, often resulting in non‐existent to small effects. The majority of these studies examined between‐person associations or average within‐person effects (Marciano et al., 2024; Valkenburg et al., 2022). This is problematic, given that recent ESM studies show that the effects of social media use on adolescents' psychosocial functioning vary from person to person (Valkenburg, Beyens, et al., 2021). Until now, evidence on person‐specific social media effects has been based on single ESM studies covering one to 3 weeks. Therefore, whether these person‐specific social media effects are changing or stable over time is still an open question. To inform media effects theories and person‐specific interventions, the present study filled this gap by investigating the stability of social media effects across two three‐week ESM studies, with 6 months and a COVID‐19 lockdown in between.

This study showed that social media effects on psychosocial functioning are person‐specific phenomena that change over time for about half to two‐thirds of the adolescents in our sample. For self‐esteem and well‐being, a low rank‐order stability was accompanied by a low mean‐level change, indicating that the social media effects changed to a different degree in sign and magnitude across adolescents. Regarding friendship closeness, a low rank‐order stability was accompanied by a positive mean‐level change. This indicates that among most, but not all, adolescents, the social media effect on friendship closeness became less negative or more positive while the COVID‐19 pandemic endured. The extent of this change differed from person to person. Specifically, whereas social media effects remained stable among 51% to 32% of the adolescents, the effects changed among the remaining 49% to 68% of the adolescents.

Overall, these findings support the idea that social media effects found at the sample level do not generalize to the individual level because they differ from person to person and change over time (Adolf & Fried, 2019; Beyens et al., 2020; Molenaar & Campbell, 2009). Therefore, both the homogeneity and stationarity assumption of ergodicity are violated. These findings further align with recent insights from personality psychology that show changes in idiographic personality profiles (Hulsmans et al., 2024). These findings suggest that future research should no longer rely on between‐person or average effects and account for their heterogeneity and stability.

### Theoretical explanations for the change in social media effects

The present study's findings may extend social media effects theories. One of the most pressing research questions regarding the impact of adolescents' social media use is why some adolescents benefit from social media use in terms of psychosocial functioning while others experience increased psychosocial problems (Valkenburg et al., 2022). The differential susceptibility to media effects model (Valkenburg & Peter, 2013) proposes that certain adolescents may be more susceptible to social media's positive or negative effects than others due to a unique combination of dispositional, developmental, and social contextual factors. Both stable and transient factors play a significant role in this model.

The effects of social media may differ from person to person due to relatively stable personal characteristics, which should make social media effects somewhat stable over time. Media effects could also depend on time‐varying situational factors, such as COVID‐19 experiences. If such time‐varying factors mainly drive social media effects, we would expect a change in social media effects. This study provides a first estimation that stable factors may account for ~ 50% of the variance in the effects of social media on well‐being and self‐esteem. The remaining 50% could be attributed to time‐varying factors or unreliability in estimations, given that social media effects were stable over time among half of the adolescents. Future research may seek a better understanding of why social media effects differ to better tailor interventions to the unique characteristics of a person, but also to the time‐specific context, content, and circumstances.

### Individual differences in the stability of social media effects

This study showed that not only do social media effects differ from person to person, but also the stability of the effects. Whereas the effects became more positive over time among some adolescents, among others, the effects became more negative. To explain this heterogeneity, we explored whether the stability of social media effects depended on gender, mean levels of psychosocial functioning and media use, and COVID‐19 experiences. After controlling for ESM 1, the social media effects on all three types of psychosocial functioning were more positive during ESM 2 among adolescents with lower psychosocial functioning during ESM 1 than among those with higher psychosocial functioning. These findings point to a poor‐get‐richer effect (Pouwels et al., 2022) that may be strengthened by the COVID‐19 pandemic that started between the two ESM studies. Previous research has shown that in‐person interactions may be relatively stressful among adolescents with low levels of psychosocial functioning (Maes et al., 2019). The COVID‐19 pandemic may have limited their in‐person interactions and increased their online interactions with peers (Keijsers & Bülow, 2021). Therefore, during COVID‐19, they may have experienced the benefits of social media (Hamilton et al., 2022; Marciano, Ostroumova, et al., 2022), where they have more time to think about their self‐presentation and interactions due to the a‐synchronicity of social media (McKenna & Bargh, 1999; Walther, 1996). Ultimately, their social media effects on psychosocial functioning may have become more positive over time.

We found that adolescents who felt that COVID‐19 had a more negative impact on their lives benefited more from social media use in terms of psychosocial functioning after the COVID‐19 lockdown than before the pandemic. While this finding might initially seem contradictory, it can be explained by considering adolescents' pre‐pandemic experiences. These adolescents likely had primarily positive in‐person interactions before the pandemic, making the role of social media less significant (Achterhof et al., 2022). During the pandemic, these adolescents may have partially compensated for the lack of inter‐person interactions during the lockdown by using social media. However, it is important to note that social media could not fully replace in‐person interactions, as these adolescents still perceived the impact of the pandemic as relatively negative.

### Avenues for future research

Although this is the first study to examine to which extent social media effects are stable over time, it also comes with limitations and suggested avenues for future research. First, we used a slightly different measure of social media use in ESM 1 than in ESM 2. Although the mean levels of social media use did not differ significantly between both studies, and there was relatively high stability over time, it may have affected the stability of the social media effects and results may reflect the lower bound of the real stability. Ideally, future research on the stability of social media effects should include measures of social media use that remain identical over time. Furthermore, it would be interesting to examine the stability of the effects of specific social media activities and content on adolescents' psychosocial functioning.

Second, the study was conducted during a highly volatile time: the COVID‐19 pandemic. The pandemic may have partly driven the suggested changes in social media effects. For example, during the first months of the pandemic, adolescents' psychosocial problems increased, and social media use may have exacerbated these problems (Draženović et al., 2023). As such, the findings of the current study may have been affected by the COVID‐19 circumstances rather than the 6‐month time interval. However, it should be noted that unlike other studies (Mansfield et al., 2022; Marciano, Viswanath, et al., 2022; von Soest et al., 2022), we did not find a decrease in adolescents' mean levels of psychosocial functioning, which could be due to the fact that ESM 2 was conducted right after the first lockdown, during which adolescents could have in‐person contact with their friends and peers again. A suggestion for future research would be to tease contextual effects (like the pandemic) and time effects apart by replicating the current findings now that COVID‐19 no longer impacts adolescents' in‐person interactions with peers.

Third, it is important to note that the here reported stability coefficients may represent an underestimation of the true stability of the social media effects (Scharkow & Bachl, 2017). We found that the posterior standard deviation of adolescents' social media effects accounted for the change in social media effects on friendship closeness but not for self‐esteem and affective well‐being. The posterior standard deviation summarizes the imprecision of an adolescent's social media effect and may be inflated due to measurement error. If measurement error is not accounted for, unsystematic errors in reporting social media use may lead to under‐estimated or spurious social media effects (Scharkow & Bachl, 2017; Schuurman & Hamaker, 2019) that may change over time. Therefore, a suggestion for future research is to account for measurement errors in estimating social media effects (Bolger et al., 2019; Schuurman & Hamaker, 2019).

Fourth, in addition to controlling for measurement error, we recommend future research to use alternative modeling strategies that better account for the dynamic nature of psychosocial functioning and that take other personal, contextual, and device‐specific factors into account (Kelty‐Stephen et al., 2023; Vanden Abeele, 2020). In our study, we investigated the stability of short‐term linear effects of social media use on psychosocial functioning. For most adolescents, these linear effects were relatively small. However, in dynamic systems (social media), effects are often nonlinear (Granic, 2005), may accumulate over time, and depend on personal, context‐specific, and device‐specific factors (Orben et al., 2024; Vanden Abeele, 2020) that have not been taken into account. Furthermore, in addition to social media use, there may be several other issues that contribute to adolescents' psychosocial functioning that we did not address, such as high pressure to perform (Stevens et al., 2024) or the climate crisis (Kramer et al., 2024).

Fifth, we examined the stability of social media effects among a relatively homogeneous sample of Dutch adolescents in Grades 8 and 9 at one secondary school. A suggestion for future research would be to examine the stability of social media effects in samples that are more diverse in terms of gender, age, socioeconomic status, ethnic background, country, and region.

Sixth, the current study examined stability over a 6‐month time interval. We still know very little about whether and to what extent social media effects also change over shorter time intervals, such as from week to week, or much longer intervals, such as from year to year. Across what time interval are social media effects stable, and among whom and when do these effects change?

### Implications for person‐specific media effects research

While idiographic methods have gained popularity in developmental psychology, communication science, and related disciplines, they have also faced criticism (Coenen, 2023; Johannes et al., 2021; Vuorre et al., 2022), which has been addressed by Valkenburg et al. (2024). The investigation of effects that are assumed to be apparent according to the teachers and parents but that remain difficult for scholars to detect has been questioned. Are these effects truly absent? Do teachers, parents, and adolescents perceive effects that do not exist? Or should we seek improved methods? We believe advancing this discussion is important for two reasons.

First, the issue holds significant societal relevance, yet the current discourse lacks a solid scientific foundation. There is ongoing societal debate regarding the negative impacts of digital media on adolescents' psychosocial functioning. While many researchers agree that on average, the effects of social media on adolescent well‐being are negligible to small, others argue that social media is a primary cause of the mental health crisis among adolescents (Haidt, 2024). Consequently, discussions persist about whether phones should be banned from schools or if social media should be restricted for children below a certain age. Understanding social media effects and their stability is essential for effectively informing this societal debate.

Second, although existing research suggests that overall, effects are minor for the average adolescent, it is crucial to recognize that the “average adolescent” is an abstraction rather than a representative reality. Our research reveals that the effects of social media are more nuanced than typically portrayed in the media. While, on average, adolescents may not be harmed by their social media use, person‐specific studies consistently indicate that a subset of adolescents experiences significant negative effects (e.g., Beyens et al., 2021; Griffioen et al., 2022; Marciano, Driver, et al., 2022; Pouwels et al., 2021a; Rodriguez et al., 2021; Valkenburg, Pouwels, et al., 2021). These findings align with adolescents' subjective experiences reported in interviews (van der Wal et al., 2024). Given that some adolescents benefit from social media while others are adversely affected, these effects may cancel each other out, resulting in relatively small overall effects or even null effects.

This study encourages our field to better align the complexity of human behaviors, as described in dynamic systems theories, with our research methods. Addressing this complexity requires a within‐person approach that is able to detect heterogeneity across individuals and stability across different timescales. Such a comprehensive approach is necessary to capture the complexity of the issue, understand why some individuals experience pronounced negative effects, determine the long‐term impact on their well‐being, and provide appropriate support for adolescents negatively affected by social media use. We recommend future research to extend the current approach by (a) employing modeling strategies that account for the dynamic nature of psychosocial functioning and (b) considering personal, contextual, and device‐specific factors (Kelty‐Stephen et al., 2023; Vanden Abeele, 2020).

### Implications for person‐specific interventions

Investigating person‐specific media effects is a promising approach for tailoring social media advice to adolescents' social media use and outcomes (Valkenburg et al., 2022). However, before the time is ripe to tailor intervention programs to specific adolescents, we need to know whether a person's social media effects are stable (Bolger et al., 2019; Bringmann, 2021). Most adolescents will sometimes, or for a given period, experience a drop in their well‐being after using social media. However, this study showed that such adverse effects seem to change for most adolescents – and one could argue that this change is a sign of adaptability and resilience (Kalisch et al., 2017). After all, what may differentiate typically developing adolescents from those who develop clinically significant psychosocial problems is the extent to which such negative media effects are dynamic and may disappear or change over time. We also identified a subgroup for whom social media effects were consistently negative (e.g., on their self‐esteem; ~ 9%). Only when social media have a constant negative effect over time could social media effects be considered as modifiable targets of interventions that may make interventions more effective than wait‐list control. Researchers and practitioners, therefore, should be aware that they may fall short if they implement person‐specific interventions based on one ESM study of only 3 weeks, as it seems essential to take the stability of social media effects into account.

## CONCLUSION

This study showed that social media effects differ not only from person to person but might also change within a person over time. Although there was some maintenance in the rank‐order stability of the social media effects on self‐esteem, the rank‐order of social media effects on affective well‐being and friendship closeness was not maintained. Individual‐level findings showed that the social media effects on self‐esteem and well‐being changed for about half of the adolescents, while the effects on friendship closeness changed for about two‐thirds of the adolescents. While COVID‐19 experiences partly explained this change, future research should be undertaken to investigate among whom, when, and why social media effects are changing versus stable.

## AUTHOR CONTRIBUTIONS

J.L.P., P.M.V., I.B., and L.K. designed the study and contributed to writing and reviewing the manuscript. J.L.P. and I.B. collected the data. J.L.P. analyzed the data.

## Data Availability

The data necessary to reproduce the analyses presented in this manuscript are publicly accessible at Figshare (Pouwels et al., 2024). The analytic code necessary to reproduce the analyses presented in this article is publicly accessible at https://osf.io/btvuw/. The materials necessary to replicate the findings presented in this article are publicly accessible at https://osf.io/btvuw/. The analyses presented here were preregistered after data collection https://osf.io/zftmv.

## APPENDIX A Exploratory analyses

**TABLE A1:** Social media use effects on psychosocial functioning in experience sampling methodology (ESM) 2 predicted by social media use (SMU) effects in ESM 1 and gender.

Coefficients	SMU effect											
	Affective well‐being ESM 2				Self‐esteem ESM 2				Friendship closeness ESM 2			
	*b*	SE	β	*p*	*b*	SE	β	*p*	*b*	SE	β	*p*
Intercept	0.10	0.01		<.001	−0.01	0.01		.062	0.10	0.01		<.001
SMU effect ESM 1^a^	0.02	0.08	.02	.761	**0.386**	**0.17**	.**26**	.**003**	−0.02	0.17	−.01	.896
Gender^b^	0.00	0.01	.02	.671	0.00	0.02	.00	.934	−0.02	0.02	−.08	.185
SMU effect ESM 1*gender	0.10	0.10	.09	.322	−0.03	0.21	.02	.842	0.16	0.21	.08	.436

*Note*: Separate models were conducted for the social media use effects on affective well‐being, self‐esteem, and friendship closeness. SMU Effect ESM 1 has been grand‐mean‐centered. *R* ^2^ was .01 for all three models.

^a^ SMU Effect ESM 1 represents the effect of social media use on psychosocial functioning during ESM 1.

^b^ Gender was dummy coded (0 = boy; 1 = girl).

**TABLE A2:** Social media use effects on psychosocial functioning in experience sampling methodology (ESM) 2 predicted by social media use (SMU) effects in ESM 1 and levels of psychosocial functioning and social media use.

Coefficients	SMU effect											
	Affective well‐being ESM 2				Self‐esteem ESM 2				Friendship closeness ESM 2			
	*b*	SE	β	*p*	*b*	SE	β	*p*	*b*	SE	β	*p*
Intercept	0.02	0.00		<.001	−0.01	0.00		.003	0.08	0.01		<.001
SMU effect ESM 1^a^	0.05	0.06	.05	.429	0.06	0.10	.04	.536	0.09	0.10	.5	.369
Level AWB/SEE/FCL ESM 1^b^	**−0.01**	**0.00**	**−.14**	.**025**	**−0.02**	**0.00**	**−.38**	**<.001**	**−0.03**	**0.01**	**−.34**	**<.001**
Level SMU ESM 1^c^	**−0.01**	**0.00**	**−.12**	.**037**	0.00	0.00	−.05	.382	**−0.02**	**0.01**	**−.22**	**<.001**
SMU effect ESM 1 * level AWB/SEE/FCL ESM 1	−0.01	0.04	−.02	.772	−0.02	0.07	−.02	.760	0.07	0.09	.05	.408
SMU effect ESM 1 * level SMU ESM 1	0.05	0.03	.09	.158	0.07	0.06	.07	.212	**0.16**	**0.07**	.**13**	.**020**

*Note*: Separate models were conducted for the social media use effects on affective well‐being, self‐esteem, and friendship closeness. All predictor variables have been grand‐mean‐centered. *R* ^2^ was .04 for the affective well‐being model, .16 for the self‐esteem model, and 12 for the friendship closeness model. Significant coefficients are depicted in bold.

^a^ SMU effect ESM 1 represents the effect of social media use on psychosocial functioning during ESM 1.

^b^ Level AWB/SEE/FCL ESM 1 represents the adolescents' person mean level of affective well‐being, self‐esteem, and friendship closeness during ESM 1.

^c^ Level SMU represents adolescents' person mean level of social media use during ESM 1.

**TABLE A3:** Social media use effects on psychosocial functioning in experience sampling methodology (ESM) 2 predicted by social media use (SMU) effects in ESM 1 and COVID‐19 experiences.

Coefficients	SMU effect											
	Affective well‐being ESM 2				Self‐esteem ESM 2				Friendship closeness ESM 2			
	*b*	SE	β	*p*	*b*	SE	β	*p*	*b*	SE	β	*p*
Intercept	0.02	0.00		<.001	−0.01	0.00		.013	0.08	0.01		<.001
SMU effect ESM 1^a^	0.10	0.05	.11	.069	**0.42**	**0.09**	.**29**	**<.001**	0.12	0.10	.07	.250
COVID negative^b^	0.00	0.00	.07	.245	0.01	0.00	.08	.150	**0.02**	**0.01**	.**15**	.**012**
COVID positive^c^	0.00	0.00	−.07	.221	0.00	0.00	−.01	.913	−0.01	0.01	−.10	.096
SMU change^d^	**−0.02**	**0.01**	**−.13**	.**030**	**−0.02**	**0.01**	**−.12**	.**046**	−0.01	0.02	−.04	.478
SMU effect ESM 1 * COVID negative	0.05	0.04	.06	.297	−0.04	0.07	−.04	.528	0.01	0.09	.01	.922
SMU effect ESM 1 * COVID positive	0.08	0.05	.10	.090	−0.01	0.08	−.01	.866	**0.24**	**0.10**	.**14**	.**020**
SMU effect ESM 1 * SMU change	0.00	0.11	.00	.968	0.25	0.18	.09	.160	0.01	0.20	.00	.974

*Note*: Separate models were conducted for the effects of social media use effects on affective well‐being, self‐esteem, and friendship closeness. All continuous predictor variables have been grand‐mean‐centered. *R* ^2^ was .06 for the affective well‐being model, .09 for the self‐esteem model, and .05 for the friendship closeness model.

^a^ SMU effect ESM 1 represents the effect of social media use on psychosocial functioning during ESM 1.

^b^ COVID negative has been measured with the question, “Is the coronavirus causing negative changes in your life?” Participants responded on a scale from 1 (not at all) to 5 (completely).

^c^ COVID positive has been measured with the question, “Is the coronavirus causing positive changes in your life?” Participants responded on a scale from 1 (not at all) to 5 (completely).

^d^ SMU change has been measured with the question, “Do you use social media differently than usual?” (0 = no; 1 = yes).

**TABLE A4:** Social media use effects on psychosocial functioning in experience sampling methodology (ESM) 2 predicted by social media use (SMU) effects in ESM and uncertainty of social media effects.

Coefficients	SMU effect											
	Affective well‐being ESM 2				Self‐esteem ESM 2				Friendship closeness ESM 2			
	*b*	SE	β	*p*	*b*	SE	β	*p*	*b*	SE	β	*p*
Intercept	0.02	0.00		<.001	−0.01	0.00		.003	0.08	0.01		<.001
SMU effect ESM 1^a^	0.10	0.05	.11	.056	**0.39**	**0.09**	.**27**	**<.001**	0.09	0.10	.05	.353
SMU effect ESM 1 (*SD*)^b^	0.35	0.20	.10	.077	−0.15	0.39	−.02	.708	**1.16**	**0.37**	.**18**	.**002**
SMU effect ESM 1 * SMU effect ESM 1 (*SD*)	−3.13	3.61	−.05	.386	6.88	7.98	.05	.390	**−12.10**	**6.01**	**−.12**	.**045**

*Note*: Separate models were conducted for the effects of social media use effects on affective well‐being, self‐esteem, and friendship closeness during ESM 2. All predictor variables have been grand‐mean‐centered. *R* ^2^ was .06 for the affective well‐being model, .07 for the self‐esteem model, and .05 for the friendship closeness model.

^a^ SMU effect ESM 1 represents the effect of social media use on psychosocial functioning during ESM 1.

^b^ SMU effect ESM 1 (*SD*) represents the posterior standard deviation of the within‐person effect of social media use on psychosocial functioning during ESM 1.
